# Synthesis of Nanoscale Zerovalent Iron (nZVI) Supported on Biochar for Chromium Remediation from Aqueous Solution and Soil

**DOI:** 10.3390/ijerph16224430

**Published:** 2019-11-12

**Authors:** Haixia Wang, Mingliang Zhang, Hongyi Li

**Affiliations:** 1 School of Water Conservancy and Environment, University of Jinan, Jinan 250022, China; stu_zhangml@ujn.edu.cn (M.Z.); lihongy10098@163.com (H.L.); 2 Shandong Provincial Engineering Technology Research Center for Ecological Carbon Sink and Capture Utilization, Jinan 250022, China

**Keywords:** zero-valent iron, biochar, hexavalent chromium, reduction

## Abstract

Maize straw biochar-supported nanoscale zero-valent iron composite (MSB-nZVI) was prepared for efficient chromium (Cr) removal through alleviating the aggregation of zero-valent iron particles. The removal mechanism of MSB-nZVI was investigated by scanning electron microscopy with energy dispersive X-ray (SEM-EDX), X-ray diffractometry (XRD), and X-ray photoelectron spectroscopy (XPS). Cr(VI) removal from aqueous solution by MSB-nZVI was greatly affected by pH and initial concentration. The removal efficiency of Cr(VI) decreased with increasing pH, and the removal kinetics followed the pseudo-second-order model. XRD patterns of MSB-nZVI before and after reaction showed that reduction and precipitation/co-precipitation (FeCr_2_O_4_, Fe_3_O_4_, Fe_2_O_3_) occurred with the conversion of Cr(VI) to Cr(III) and Fe(0) to Fe(II)/Fe(III). The produced precipitation/co-precipitation could be deposited on the MSB surface rather than being only coated on the surface of nZVI particles, which can alleviate passivation of nZVI. For remediation of Cr(VI)-contaminated saline–alkali soil (pH 8.6–9.0, Cr 341 mg/kg), the released amount of Cr(VI) was 70.7 mg/kg, while it sharply decreased to 0.6–1.7 mg/kg at pH 4.0–8.0, indicating that the saline–alkali environment inhibited the remediation efficiency. These results show that MSB-nZVI can be used as an effective material for Cr(VI) removal from aqueous solution and contaminated soil.

## 1. Introduction

Chromium is a highly toxic contaminant in industrial wastewater that is discharged from metallurgy, leather tannery, electroplating process, and dyeing, etc. Hexavalent chromium (Cr(VI)) and trivalent chromium (Cr(III)) are the main valence states present in the wastewater and contaminated soil, and Cr(VI) is much more soluble, mobile, and toxic than Cr(III) [1]. The Cr(VI) species including chromate (CrO_4_^2−^, HCrO_4_^−^) and dichromate (Cr_2_O_7_^2−^) can cause serious health issues. According to the World Health Organization (WHO) guidelines and Chinese standards (GB5749-2006), less than 0.05 mg/L Cr(VI) is acceptable for the required quality of drinking water [2,3]. Thus, the efficient removal of Cr(VI) from wastewater in recent years has become an urgent environmental issue.

Nanoscale zero valent iron (nZVI) refers to iron with a size below 100 nm, which has great application potential in the field of environmental remediation because of its high surface activity and strong reduction ability. In recent years, nZVI has been successfully used for the treatment of many contaminants from wastewater and contaminated soil, including inorganic pollutants (e.g., Cr(VI), As(V), Cu^2+^, NO_3_^−^), and organic pollutants (e.g., Polychorinated biphenyls (PCBs), Dichlorodiphenyltrichloroethane (DDT), organic dyes, halogenated hydrocarbons) [4,5,6,7]. However, there are many limiting factors for nZVI application. There is always a danger of prior oxidation for nZVI particles, which happens on the surfaces of the nanoparticles and then makes them non-reactive. The transport of nZVI particles is usually very difficult when added into soil. In addition, nZVI particles have a tendency to aggregate, which can result in a decrease in reactivity and removal efficiency for contaminates. In order to overcome this drawback, many supporting materials (such as activated carbon and clay minerals) have been used to prevent nZVI aggregation and increase the reactivity [8]. Biochar is a high-carbon, fine-grained, and stable material that is produced via pyrolysis of biomass feedstocks in limited oxygen environment or absence of oxygen. In recent years, biochar has been successfully used for carbon sink, wastewater treatment, and soil remediation. Due to its high stability and large specific surface area, biochar is one of the most promising supporting materials for nZVI [9]. Meanwhile, biochar demonstrated high adsorption capacity due to its abundant surface functional groups and large specific surface area. Some raw and modified biochars were used for the remediation of wastewater and contaminated soil [1,10,11,12,13]. Biochar could perform better if combined with nZVI for the remediation of Cr(VI) from aqueous solution and soil. Shang et al. (2017) synthesized nZVI particles supported on herb-residue biochar and it exhibited a high removal efficiency for Cr(VI) removal from aqueous solution [9]. Zhou et al. (2014) prepared a novel sorbent through combing biochar, chitosan, and nZVI, which showed the enhanced ability to remove Pb, Cr(VI), As(V), phosphate, and methylene blue from aqueous solution [14]. Su et al. (2016) prepared biochar-supported nanoscale zero-valent iron (nZVI-BC) for enhanced transport and in situ remediation of Cr(VI) in soil, and the remediation reduced the phytotoxicity of Cr(VI) [15].

Most of the literature only focuses on Cr(VI), and there are few studies on both total Cr and Cr(VI) removal by nZVI supported on biochar. A large amount of maize straw is produced every year in northern China, and some has not been effectively used, which can result in a great waste of resources. Conversion of maize straw into biochar is an environmentally friendly way to utilize maize straw. It is important to reveal the exact removal mechanism of Cr(VI) by biochar-supported nZVI for wastewater and soil remediation for large-scale practice.

In this study, to enhance the reactivity of nZVI particles, maize straw biochar (MSB) was investigated to support nZVI particles for Cr(VI) removal from aqueous solution and contaminated saline–alkali soil. The objectives of this study are to (1) synthesize and characterize MSB-supported nZVI composites, (2) analyse the removal efficiency of Cr(VI) and total Cr from aqueous solution and contaminated saline–alkali soil by MSB-nZVI in different experimental conditions, and (3) reveal the removal mechanisms to provide basic information for the application of this synthetic composite.

## 2. Materials and Methods

### 2.1. Synthesis of MSB-nZVI

Maize straw samples were collected in one farm in Shandong province, China, and pyrolyzed at 500 °C for 2 h in a limited-oxygen environment (without nitrogen gas flow), using a muffle furnace (SX_2_-5-12, Longkou Xianke Instrument company, Shandong, China). After cooling, MSB samples were washed with deionized water several times and then dried for the preparation of MSB-supported nZVI composite (MSB-nZVI). MSB-nZVI samples were synthesized as follows: 1 g MSB sample was added into the solution (5 g analytical grade FeSO_4_·7H_2_O dissolved in 500 mL deionized water) for 24 h at room temperature (20–25 °C). The solution was purged with nitrogen gas for 30 min to remove dissolved oxygen, and then 100 mL sodium borohydride solution (analytical grade NaBH_4_, 0.5 M) was added dropwise into the suspension with nitrogen gas bubbles. The solid products were separated from the solution by vacuum filtration, washed several times with ethanol, and then vacuum dried at 60 °C for 24 h [9,16]. The MSB-nZVI samples were stored in a sealed plastic (PE) bag to prevent oxidization at 4 °C.

### 2.2. Sample Characterizations

The specific surface areas of MSB and MSB-nZVI were analyzed using an ASAP 2020 surface area and porosity analyzer (Micromeritics, Norcross, GA, USA). A scanning electron microscope (SEM, Quanta FEG 250, Hillsboro, OR, USA) coupled with an energy dispersion spectrometer (EDX, X-Max50, Oxford, UK) was used to observe surface morphology and chemical elements of MSB-ZVI before and after reaction with Cr(VI). The surface functional groups of the samples were examined by a Nicolet 380 FTIR spectrometer (Thermo Scientific, Waltham, MA, USA). Mineralogical characterization of MSB-nZVI before and after reaction with Cr(VI) was performed by a powder X-ray diffractometer (XRD, Bruker D8 ADVANCE, Berliln, Germany).

### 2.3. Remediation of Cr(VI) and Total Cr from Aqueous Solution

#### 2.3.1. Effect of pH, Initial Concentration, and Contact Time

To determine the effect of solution pH on Cr(VI) and total Cr removal by MSB-nZVI, 0.1 g sample and 25 mL Cr (VI) solution (0.48, 0.96, 1.92 mM) were mixed without shaking for 48 h. Considering electroplating wastewater containing high concentration of Cr(VI), the concentrations of 0.48, 0.96, and 1.92 mM (25, 50, and 100 mg/L) were chosen for the experiments. The solution pH was adjusted in the range of 2.0–8.0 by addition of NaOH or HNO_3_ solution. The solution pH was measured with a pH meter (Leici PHS-3C, Shanghai, China). For the effect of initial concentration, 0.1 g MSB-nZVI sample was added into a series of PET plastics bottles containing 25 mL Cr(VI) solution with different concentrations at pH 2.5. The mixtures were centrifuged and filtered through 0.45 μm filter paper for Cr(VI) and total Cr concentration analysis after mixing for 48 h. For the effect of contact time, 2 g MSB-nZVI sample was mixed with 500 mL Cr(VI) solutions (1.92 mM) at pH 2.5. The samples were withdrawn at different time intervals.

The concentration of Cr(VI) was determined by the standard 1, 5-diphenylcarbazide spectrophotometric method (GB7647-87) using a UV-Vis spectrophotometer (UV-2550 spectrophotometer, Shimadzu, Japan) at 540 nm. The detection limit of Cr(VI) was 0.004 mg/L. The concentration of total Cr was determined by atomic absorption spectroscopy (AAS, AA-7000 model spectrometer, Shimadzu, Japan).

#### 2.3.2. Methods to Reveal Cr(VI) Removal Mechanism

In order to reveal the mechanisms of Cr(VI) removal by MSB-nZVI, XRD analysis was performed to analyze the crystallinity formed on the surface of MSB-nZVI after reaction with Cr(VI). The surface morphological structure and elemental composition before and after Cr(VI) removal were characterized by SEM-EDX. The valence state of Cr and Fe on the surface of MSB-nZVI before and after reaction with Cr(VI) was determined by X-ray photoelectron spectroscopy (XPS, Thermo ESCALAB 250XI, MA, USA), in order to reveal the conversion of valence state of Cr and Fe during the reaction.

### 2.4. Remediation of Cr(VI)-Contaminated Soil

#### 2.4.1. Analytical Methods for Soil Characterizations

The Cr(VI)-free soil sample was collected in Binzhou, Shandong province, China. The pH of the soil sample was measured with a pH meter (Leici PHS-3C, Shanghai, China) (soil/water (1:2.5)) [17]. The water-soluble salt content was measured by residue drying method (soil/water (1:5)) [18]. Cr(VI)-contaminated soil samples were prepared as follows: K_2_Cr_2_O_7_ solution (500 mL) at the desired concentration was added into air-dried soil (500 g), and the mixture was mixed thoroughly and air-dried to a constant weight.

#### 2.4.2. Remediation Experiments of Cr(VI)-Contaminated Soil

The soil remediation tests were conducted with 5 g soil and 50 mL deionized water (soil-to-solution ratio of 1:10) at room temperature (20–25 °C) for the water-soluble released amounts of Cr(VI) or total Cr. In order to study the effect of pH on Cr(VI) remediation, the mixture was adjusted to be 4, 5, 6, 7, 8, and 9, respectively. To study the dosage of MSB-nZVI on released amounts of total Cr and Cr(VI), the soil samples were mixed with MSB-nZVI at pH 7.0, and the dosages of MSB-nZVI were 0, 2, 5, 10, 20, and 40 g/kg, respectively. To investigate the effect of reaction time on Cr(VI) remediation, the soil samples were mixed with MSB-nZVI (40 g/kg dosage) at pH 7.0. Samples were withdrawn at 1, 2, 4, 6, 8, 12, 24, 36, and 48 h, respectively. All the soil experiments were conducted in triplicate, and the average values were presented.

## 3. Results and Discussion

### 3.1. Characterization of MSB-nZVI

**Figure 1:**
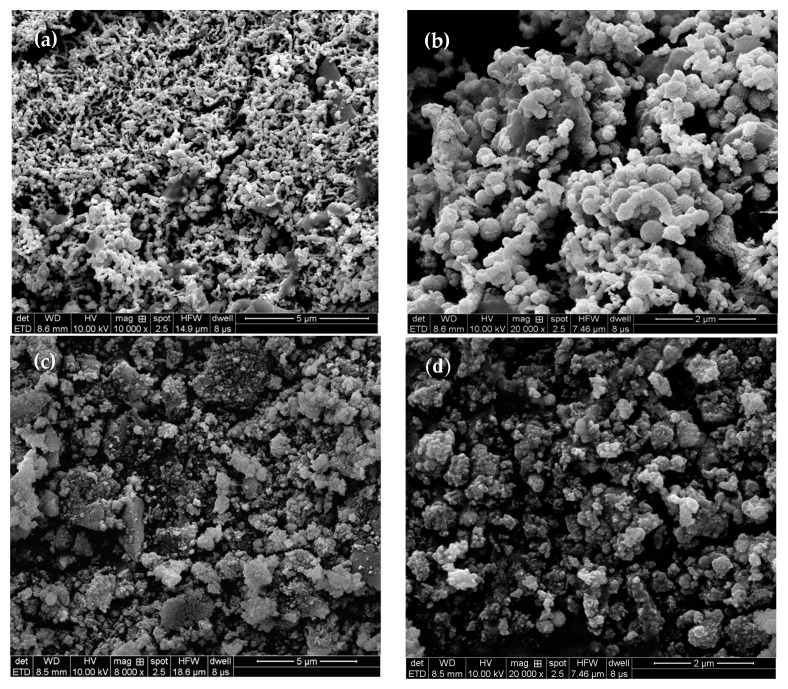
SEM images of maize straw biochar-supported nanoscale zero-valent iron composite (MSB-nZVI) before (**a**,**b**) and after reaction with Cr(VI) (**c**,**d**).

**Figure 2:**
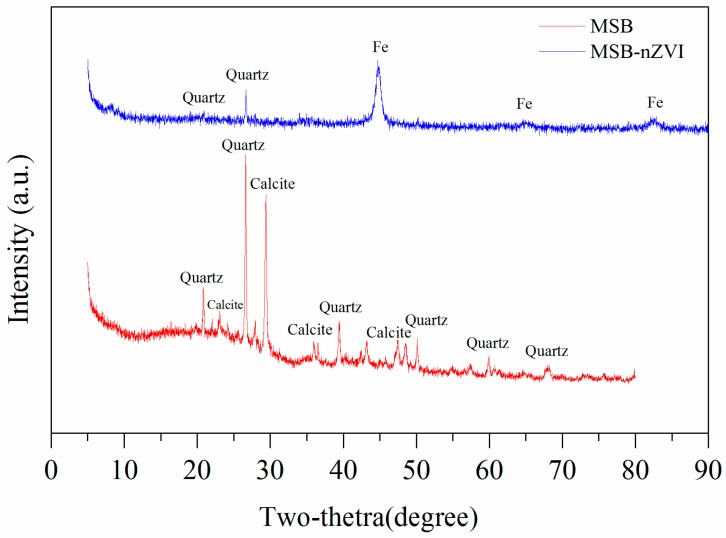
X-ray diffractometer (XRD) patterns of MSB and MSB-nZVI.

The surface morphologies of MSB-nZVI before and after reaction with Cr(VI) are shown in Figure 1a–d. Many spherical particles (ZVI) with sizes ranging from 40 to 100 nm are evenly attached to the surface of MSB. The surface of the MSB-nZVI after reaction with Cr(VI) appears to be coarser with rough aggregates on the surface, which could be related to the corrosion of nZVI particles and chromium/iron oxides via redox reactions. The specific surface area of MSB was 24.5 m^2^/g and it increased to 30.5 m^2^/g for MSB-nZVI. As shown in Figure 2, XRD patterns (without smooth treatment) showed that quartz and calcite were the main minerals for MSB, and the clear characteristic peaks of ZVI (2θ = 44.7, 64.9, and 82.2) for MSB-nZVI.

**Figure 3:**
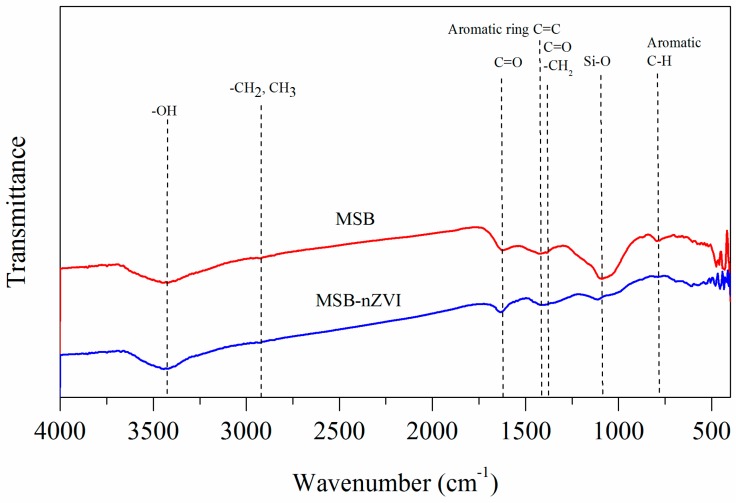
Fourier transform infrared (FTIR) spectra of MSB and MSB-nZVI.

The Fourier transform infrared (FTIR) spectra (Figure 3) for MSB showed that the broad band around 3421 cm^−1^ was related to the -OH vibration, indicating hydroxyl groups and adsorbed water existing on the surface of MSB. The bands around 2923 cm^−1^ correspond to the −CH_2_ and −CH_3_ group of long-chain aliphatic components. The characteristic bands at around 1620 cm^−1^ and 1097 cm^−1^ correspond to C=O and Si–O stretching vibrations, respectively. The band at around 781 cm^−1^ is attributed to aromatic C–H [19]. The intensity of the typical bands all decreased for MSB-nZVI due to the surface of MSB being coated by nZVI particles.

### 3.2. Cr(VI) Removal from Aqueous Solution

#### 3.2.1. Effect of pH

**Figure 4:**
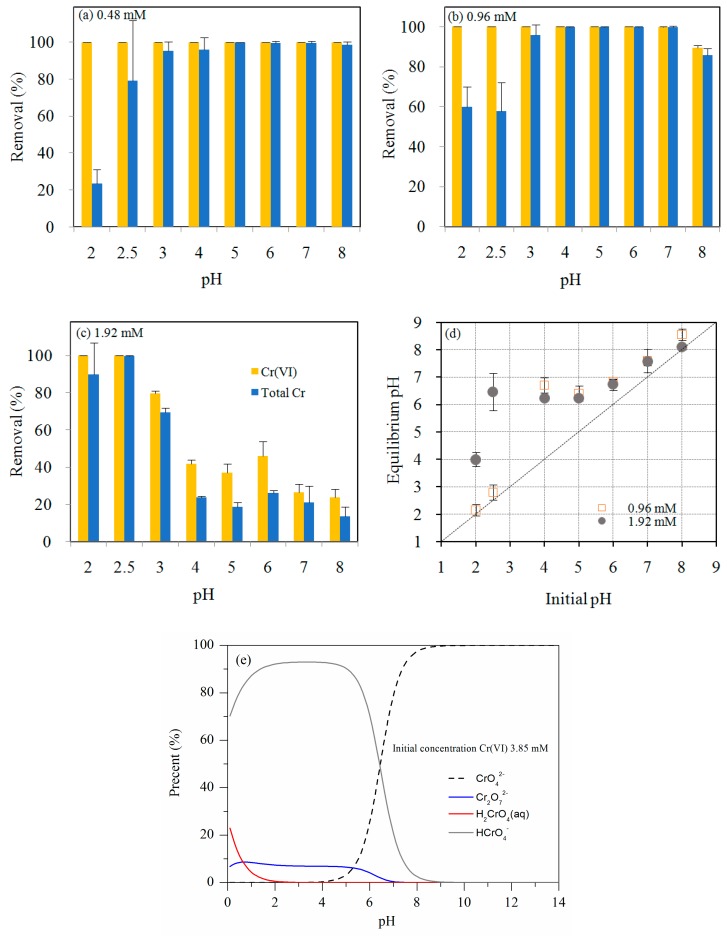
The effect of pH on Cr removal efficiency (**a**,**b**,**c**); the equilibrium pH after reaction with Cr(VI) at different initial pH (**d**); the speciation plot for Cr(VI) in aqueous solution as a function of pH (Cr(VI) 3.85 mM) (**e**).

The effect of pH on the removal efficiencies of Cr(VI) and total Cr by MSB-nZVI is shown in Figure 4. For the initial Cr(VI) concentration of 0.48 mM, more than 99.9% of Cr(VI) was reduced at pH 2.0–8.0, and the removal efficiency of total Cr increased from 23.5% to more than 99.9% with pH increasing from 2.0 to 8.0. This is because increasing pH was favorable for the converted Cr(III) ions precipitated as Cr(III) hydroxides and/or the form of mixed Fe/Cr (oxy) hydroxides (Equations (1)–(3)). For the initial concentration of Cr(VI) 0.96 mM, the removal efficiency of Cr(VI) decreased from more than 99.9% to 89.4% with pH increasing from 2.0 to 8.0. The removal of total Cr increased from 60% to 99.8% with pH increasing from 2.0 to 7.0, and then decreased to 85.7% at pH 8.0. For Cr(VI) 1.92 mM, the removal efficiency of Cr(VI) decreased from more than 99.9% to 23.8% with pH increasing from 2.0 to 8.0. The removal of total Cr increased from 89.7% to 99.9% with pH increasing from 2.0 to 2.5, and then decreased to 13.5% at pH 8.0. The removal efficiencies of Cr(VI) were significantly higher at pH 2.0–2.5 than those observed at different pH values (*p* < 0.05), indicating that the solution pH greatly affected the removal performance of Cr(VI) by MSB-nZVI. Acidic environment is favorable for Cr(VI) removal according to the following reactions [16,20]:

(1) $$2{\mathrm{HCrO}}_{4}{}^{-}+3\mathrm{Fe}(0)+14H^{+}\to 2{\mathrm{Cr}}^{3+}+3{\mathrm{Fe}}^{2+}+8H_{2}O$$

(2) $${\mathrm{HCrO}}_{4}{}^{-}+3{\mathrm{Fe}}^{2+}+7H^{+}\to 2{\mathrm{Cr}}^{3+}+3{\mathrm{Fe}}^{3+}+4H_{2}O$$

(3) $$(1-x){\mathrm{Fe}}^{3+}+x{\mathrm{Cr}}^{3+}+3H_{2}O\to {\mathrm{Cr}}_{x}{\mathrm{Fe}}_{1-x}{\left(\mathrm{OH}\right)}_{3}+3H^{+}$$

For initial concentration of Cr(VI) 1.92 mM, the equilibrium pH after reaction increased to 4.0, 6.2, and 7.6 for the initial pH 2.0, 5.0, and 7.0, respectively. The larger degree of pH increase for initial pH 2.0 further suggested that the oxidation–reduction reaction between Cr(VI) and Fe(0)/Fe(II) intensified at low pH. Meanwhile, the electrostatic attraction between MSB and Cr(VI) could be enhanced at lower pH, which consequently facilitated the adsorption of Cr(VI) on MSB-ZVI and promoted the reaction between Fe(0) and Cr(VI).

The removal efficiency of total Cr at pH 2.0–2.5 was 23.5–79.2%, 57.9–60%, and 89.7–99.9% for 0.48, 0.96, and 1.92 mM, respectively. It showed that the removal efficiency of total Cr increased with initial Cr(VI) concentration increasing from 0.48 to 1.92 mM. At pH 2.0–2.5, more than 99.9% of Cr(VI) was reduced for the initial Cr(VI) concentration of 0.48, 0.96, and 1.92 mM. Thus, the removal of total Cr is dependent on Cr(VI) reduction to Cr(III) and subsequent Cr(III) precipitation removal, which is mainly controlled by solution pH. As shown in Figure 4d, the equilibrium pH after reaction for Cr(VI) 0.96 mM (initial pH 2.0–2.5) only increased to 2.2–2.8, while the equilibrium pH for Cr(VI) 1.92 mM (initial pH 2.0–2.5) increased sharply to 4.0–6.5, which was favorable for the converted Cr(III) precipitation and the removal of total Cr.

#### 3.2.2. Effect of Initial Concentration

**Figure 5:**
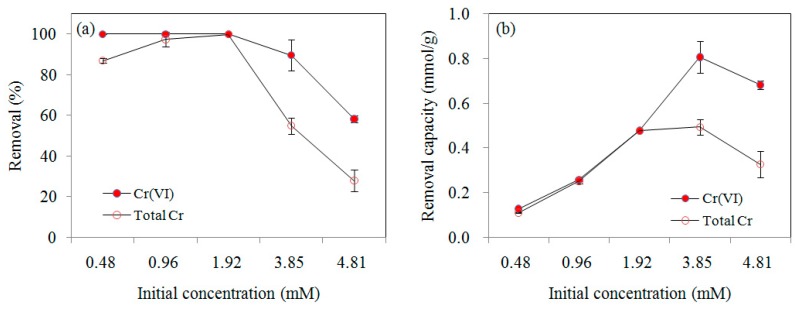
Cr(VI) removal efficiency (**a**) and capacity (**b**) by MSB-nZVI (dosage 4 g/L, pH 2.5) at different Cr(VI) initial concentrations.

The effect of initial Cr(VI) concentration on its removal efficiency was investigated in the range of 0.48–4.81 mM at pH 2.5. As shown in Figure 5, at an initial Cr(VI) concentration of 0.48–1.92 mM, the removal efficiency of Cr(VI) was over 99.9%, and it decreased to 89.6% and 58.3% for 3.85 and 4.81 mM, respectively. At an initial Cr(VI) concentration of 0.48, 0.96, 1.92, and 3.85 mM, about 0.13, 0.26, 0.48, and 0.81 mmol/g of the Cr(VI) were removed within 48 h, respectively. It appeared that the removal capacity of Cr(VI) significantly increased with increasing initial Cr(VI) concentration. However, when the initial Cr(VI) concentration increased to 4.81 mM, the unit removal capacity decreased to 0.68 mmol/g. According to the theoretical calculation, the nZVI supported by MSB in the solution was enough to reduce Cr(VI) for all the treatments (0.48–4.81 mM). High Cr(VI) concentrations caused intensive oxidation of nZVI and the rapid formation of a compact passivation layer of Cr/Fe oxides/hydroxides coated on the MSB-nZVI surface, so that the subsequent redox process was inhibited [21].

#### 3.2.3. Effect of Contact Time

**Figure 6:**
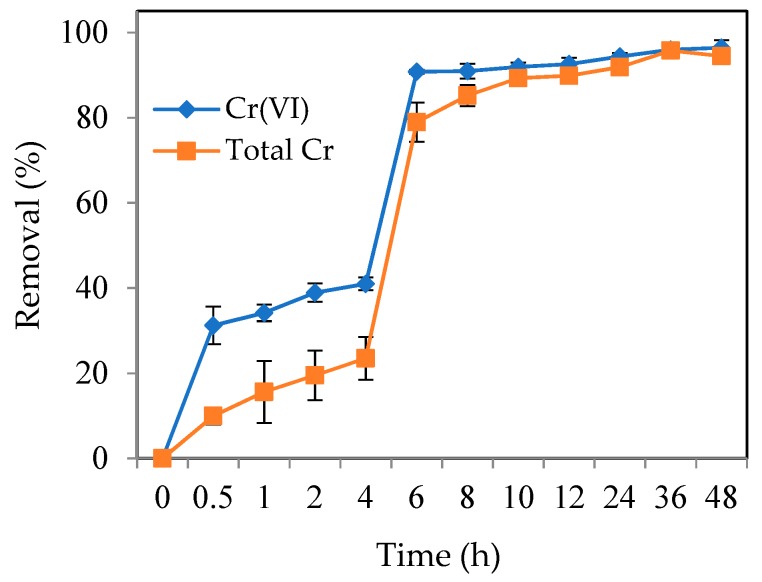
Removal efficiency of Cr(VI) and total Cr with contact time (dosage 4 g/L, initial concentration 1.92 mM Cr(VI), pH 2.5).

The effect of contact time on the removal efficiencies of Cr(VI) and total Cr is shown in Figure 6. It shows typical biphasic kinetics with rapid removal at 6 h, followed by a slower one. During the first 6 h, the removal efficiencies of Cr(VI) and total Cr increased rapidly to 90.8% and 78.9%, respectively, and then removal equilibrium was reached within 48 h, equivalent to 96.4% and 94.5%. The pseudo-first-order model and the pseudo-second-order model were used to study the adsorption kinetics process. The calculated removal capacity was closer to the experimental value for the pseudo-second-order model than for the pseudo-first-order model. The kinetic data fitted the pseudo-second-order model better than the pseudo-first-order model. It indicated that Cr(VI) removal by MSB-nZVI was controlled by a chemical process and that adsorption, reduction, and coprecipitation could occur during Cr(VI) and total Cr removal.

#### 3.2.4. Removal Mechanisms

**Figure 7:**
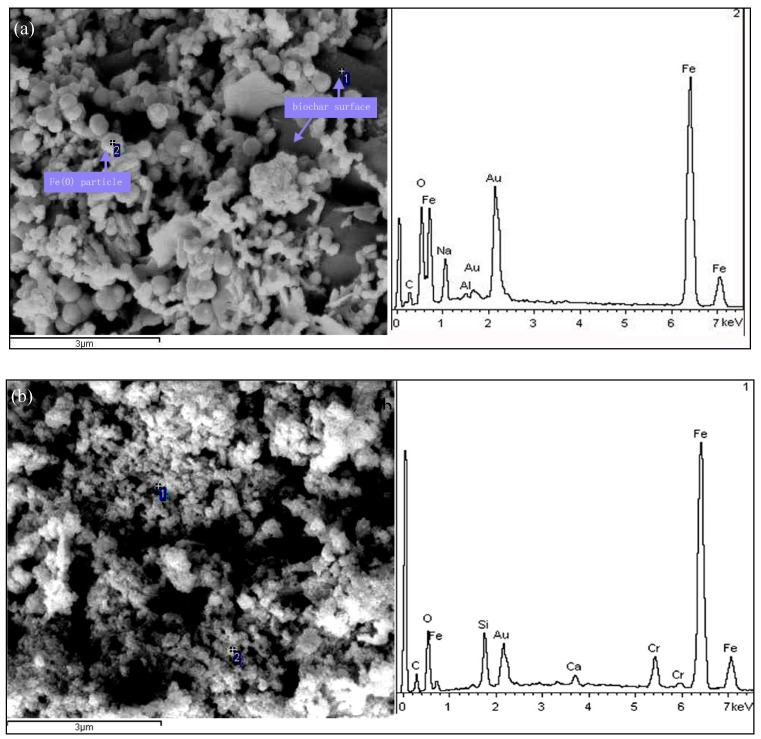
SEM-EDX analysis of before (**a**) and after (**b**) reaction with Cr(VI) 1.92 mM.

The SEM images of MSB-nZVI before and after reaction with Cr(VI) are shown in Figure 7. For the MSB-nZVI before reaction with Cr(VI), iron particles were distributed on the surface of MSB in the shape of a sphere. The EDX spectra showed the presence of Cr and Fe on the surface of MSB-ZVI after reaction with Cr(VI), and the spherical nZVI particles disappeared with rough aggregates on the surface, which could be related to the corrosion of the nZVI particles and chromium oxides via redox reactions.

**Figure 8:**
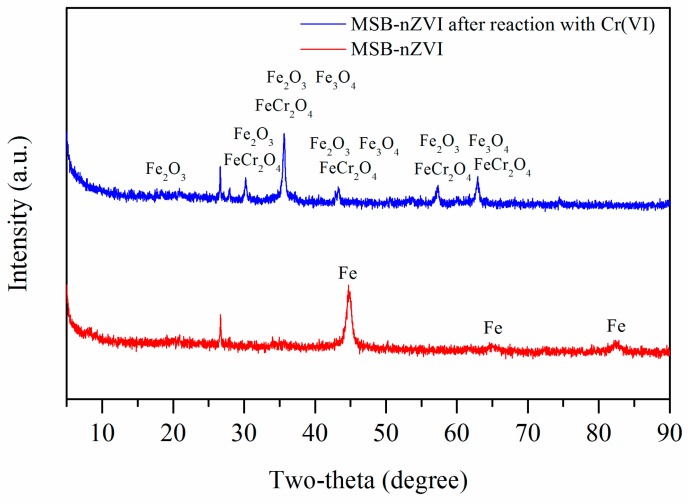
XRD patterns of MSB-nZVI before and after reaction with Cr(VI) 1.92 mM.

In order to reveal the mechanism of Cr(VI) removal by MSB-nZVI, XRD analysis was performed to analyze the possible mineral precipitates. The minerals of MSB-nZVI before and after reaction are shown in Figure 8. The XRD patterns of MSB-nZVI before reaction with Cr(VI) showed a clear characteristic peak of Fe(0) (2θ = 44.7, 64.9, and 82.2), which disappeared after the reaction with Cr(VI) [22,23,24]. The XRD patterns of MSB-nZVI after reaction might indicate the presence of Cr_2_FeO_4_, Fe_3_O_4_, and Fe_2_O_3_, which demonstrated the redox reactions between nZVI particles and Cr(VI). Cr(VI) was reduced to Cr(III) and nZVI was converted to Fe(II) and Fe(III).

**Figure 9:**
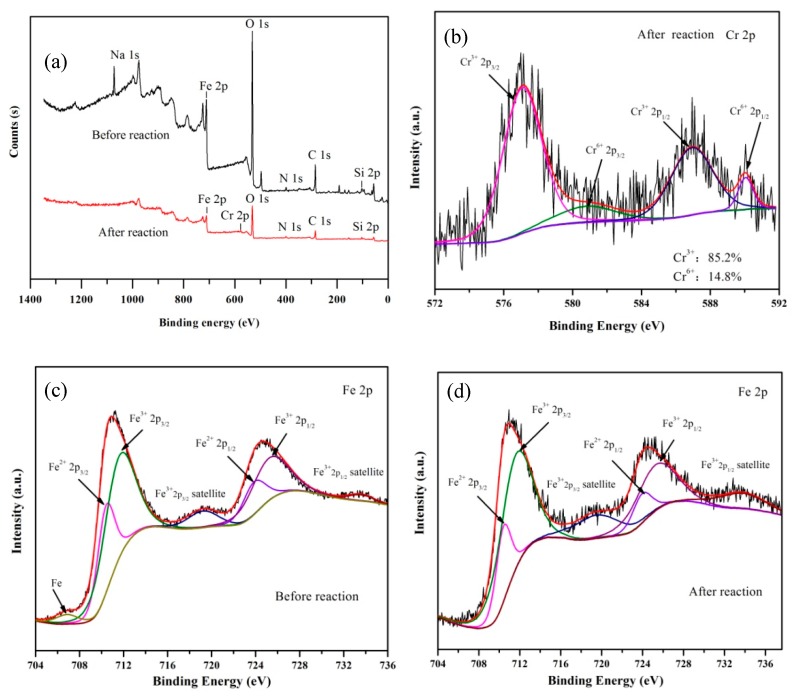
X-ray photoelectron spectroscopy (XPS) of the MSB-nZVI before and after the reaction: Full survey (**a**); Fe 2p after reaction (**b**); Cr 2p before reaction (**c**); and Cr 2p after reaction (**d**).

The detailed XPS spectra of the MSB-nZVI before and after Cr(VI) reaction in aqueous solution are shown in Figure 9. The full survey peaks of Cr at binding energy 578 eV appeared after the reaction, indicating that Cr existed on the surface of the MSB-nZVI and participated in the removal processes (Figure 9a). In addition, Cr(VI) and Cr(III) coexisted in the spectra of Cr 2p after the reaction. As shown in Figure 9b, the two peaks at 586.9 eV (Cr 2p1/2) and 577.0 eV (Cr 2p3/2) were assigned to insoluble Cr(III) oxygen/hydroxide (Cr_2_O_3_/Cr(OH)_3_) (accounting for 85.2% of total Cr according to peak area semi-quantitative analysis). The peaks at 580 eV (Cr 2p3/2) and 590 eV (Cr 2p1/2) corresponding to Cr(VI) accounted for 14.8% of total Cr. It is evident that most of the Cr adsorbed on the surface of MSB-nZVI was reduced to Cr(III) (85.2%) with zero-valent iron as electron donors [25], with only 14.8% remaining as Cr(VI) (Figure 9b). Therefore, the reduction of Cr(VI) to insoluble Cr(III) was the primary Cr(VI) removal mechanism, and the adsorption process of Cr(VI) was involved in Cr(VI) removal [26].

For the original MSB-nZVI, the peaks at binding energies of 725.2 eV (Fe 2p1/2) and 711.5 eV (Fe 2p3/2) could be assigned to Fe(III) in Fe_2_O_3_, and the two peaks at binding energies of 710.4 eV (Fe 2p3/2) and 723.8 eV (Fe 2p1/2) can be attributed to Fe(II) in Fe_3_O_4_. The peaks at 706.9 eV correspond to Fe(0), which only accounts for 2.9% of Fe species (Figure 9c–d). This is because the iron oxides layer (Fe_2_O_3_ and Fe_3_O_4_) existed on the nZVI particle surface, and XPS technique is only used for surface detection (only 2–5 nm probing depth). After the Cr(VI) reaction, the Fe(0) peaks in the spectra totally disappeared, indicating that the Fe(0) was involved in the reaction between nZVI and Cr(VI). Further analysis of the binding energy of Fe 2p suggested that Fe_2_O_3_, Fe_3_O_4_, and FeCr_2_O_4_ could be the main reaction product of MSB-nZVI after reaction with Cr(VI) [27,28].

**Figure 10:**
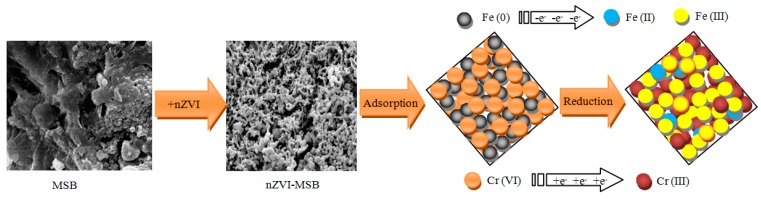
Cr(VI) removal mechanisms by MSB-nZVI.

Based on the above analysis of EDX, XRD, and XPS, the proposed mechanism of Cr(VI) removal by MSB-nZVI is shown in Figure 10. Adsorption, reduction, and precipitation/co-precipitation could be involved in the removal process of Cr(VI) by MSB-nZVI. Cr(VI) was adsorbed onto the material surface and reduced to Cr(III) by oxidizing Fe(0) to Fe(II) and Fe(III). Subsequently, the converted Cr(III) ions could form the co-precipitates as FeCr_2_O_4_ (Equation (3)). One portion of the generated Fe(III)/Cr(III)(oxy) hydroxides could be coated onto the surface of MSB because of the large surface area, which can alleviate passivation of nZVI. Compared with bare nZVI particles, the existence of biochar can enhance the reduction of nZVI due to effective dispersion of the nZVI nanoparticles [9,15,16,21,22,23,28,29,30].

### 3.3. Remediation of Cr(VI)-Contaminated Soil

#### 3.3.1. Characterization of Soil Sample

The soil belongs to sandy loam according to international standards for soil texture classification. The pH of soil sample was 8.6–9.0. The water-soluble salt content was 2.8 g/kg, according to residue drying method (soil/water (1:5)), indicating that it belonged to saline–alkali soil. The Cr(VI) content of contaminated soil was 341 mg/kg. The soil sample was ground and passed through a 2 mm sieve for the remediation experiments.

#### 3.3.2. Effect of MSB-nZVI Dosage

**Figure 11:**
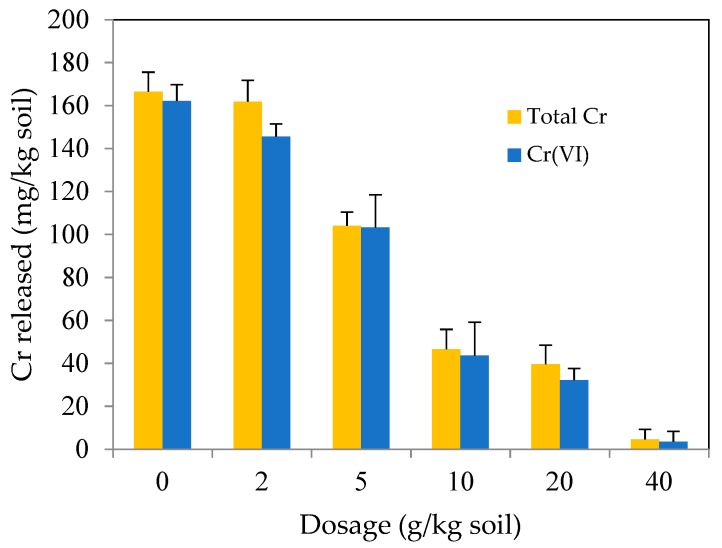
The effect of MSB-nZVI dosage on the released amount of Cr from soil.

The effect of MSB-nZVI dosage on the released amounts of Cr(VI) and total Cr is shown in Figure 11. The released amounts of Cr(VI) and total Cr from soil decreased as the MSB-ZVI dosage increased, due to the increased reactive sites. When MSB-nZVI dosage was 0, 2, 5, 10, 20, 40 g/kg, the released amount of Cr(VI) was 162.1, 145.5, 103.2, 43.6, 32.2, and 3.5 mg/kg from contaminated soil within 48 h, and the released amount of total Cr was 166.5, 161.8, 104.1, 46.5, 39.6, and 4.5 mg/kg, respectively. This was mainly attributable to the increased surface area and active sites. Similarly, a dramatic decrease in total Cr was also observed, and its downtrend was close to that of Cr(VI), indicating that most of the reduced Cr(III) had been transformed to the solid phase. The dosage 40 g/kg was larger than that reported in literature. For example, Su reported that the optimum dosage of the nZVI–Biochar composite was 8 g/kg when the remediation time was 15 d [29]. This is probably due to the less remediation time (2 d) and relatively lower surface area of MSB (24.5 m^2^/g) in this study, compared to literature.

#### 3.3.3. Effect of pH

**Figure 12:**
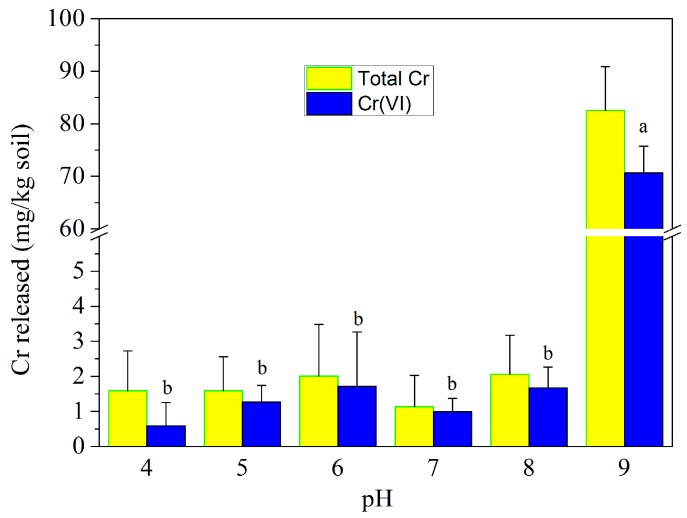
The effect of pH on the released amount of Cr from contaminated soil. The error bars marked with different letters indicate significant differences (*p* < 0.05) for Cr(VI) release at different pH.

The effect of soil pH on the released amount of Cr(VI) and total Cr is shown in Figure 12. The released amount of Cr(VI) and total Cr decreased with soil pH increasing from 2.0 to 8.0, though they were in the range of 0.6–1.7 mg/kg and 1.1–2.1 mg/kg at pH 4.0–8.0, respectively. Compared to Cr(VI)-contaminated saline–alkali soil (pH 8.6–9.0), the immobilization efficiency reached over 99% with the remediation of MSB-nZVI at adjusted pH 4.0–8.0, and the release of Cr(VI) was significantly inhibited (*p* < 0.05). However, when soil pH increased to 9.0, the amounts of Cr(VI) and total Cr released from soil sharply increased to 70.7 mg/kg and 82.6 mg/kg, respectively. An acidic environment was favorable for Cr(VI) reduction to Cr(III), and the remediation performance for alkaline soil was relatively poor [30].

#### 3.3.4. Remediation Kinetics

**Figure 13:**
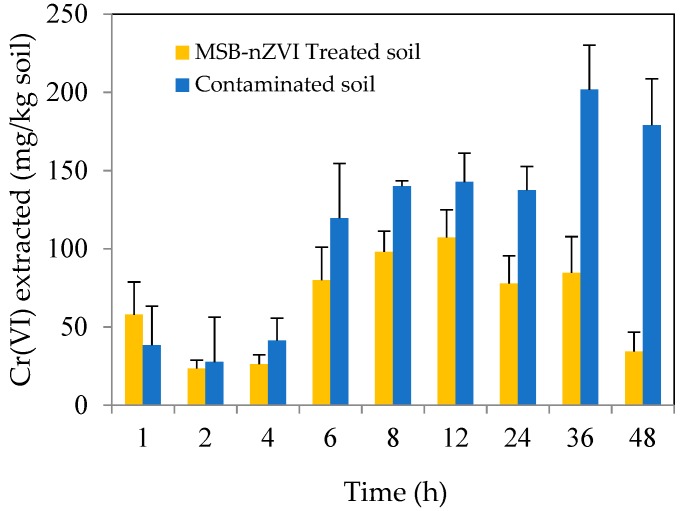
Variation of the released Cr(VI) from contaminated soil and treated soil.

The effect of contact time on Cr(VI) release from contaminated soil is shown in Figure 13. The released amount increased from 38.3 mg/kg to 201.7 mg/kg, with contact time increasing from 1 h to 48 h for the contaminated soil. Meanwhile, for the MSB-nZVI treated soil, the released amount decreased from 57.9 mg/kg to 34.4 mg/kg, with contact time increasing from 1 h to 48 h. Compared with contaminated soil, the released amount of Cr(VI) for MSB-nZVI treated soil significantly decreased due to the remediation of MSB-nZVI.

## 4. Conclusions

In this study, maize straw biochar supported nanoscale zero-valent iron composite (MSB-nZVI) was synthesized for efficient Cr removal. Cr(VI) removal from aqueous solution by MSB-nZVI was dependent on pH and initial concentration. Adsorption, reduction, and precipitation/co-precipitation were involved in the Cr(VI) removal process by MSB-nZVI, and the formed precipitate/co-precipitates (FeCr_2_O_4_, Fe_3_O_4_, and Fe_2_O_3_) were partially coated on the surface of MSB, which can alleviate passivation of nZVI. For the remediation of Cr(VI)-contaminated saline–alkali soil (pH 8.6–9.0), the immobilization efficiency of Cr(VI) was significantly inhibited, while it could reach over 99% with MSB-nZVI in adjusted pH 4.0–8.0. These results show that MSB-nZVI can be used as an effective material for Cr(VI) removal from aqueous solution and contaminated soil.
